# Matteucci’s Lectures

**Published:** 1848

**Authors:** 


### ARTICLE VI.

*Lectures on the Physical Phenomena of Living Beings.* By CARLO MATTEUCCI, Professor in the University of Pisa. Translated under the superintendence of JONATHAN PAREIRA, M.D., F.R.S., Vice President of the Royal Medical and Chirurgical Society. Philadelphia: Lea & Blanchard. 1848. 12mo. pp. 388.

The author of the work with the above title gives evidence, in these lectures, of an enthusiastic and discriminating mind engaged most laboriously in investigations upon one of the most intricate subjects—that of determining to what extent and in what instances the vital phenomena can be shown to be dependant upon physical causes.

The popularity of these lectures is evident, from the fact that two editions had been published in Italy and one in France previous to its translation into English.

Its republication in this country, places it within the power of all to secure for themselves one of the most interesting works recently published. No modern production has afforded us more satisfaction and pleasure in the perusal; we therefore unhesitatingly advise all such as have not already had an opportunity of doing so, to add it to their library. H.

